# Supplementary material for: Effects of Marinades Prepared from Food Industry By-Products on Quality and Biosafety Parameters of Lamb Meat
Source: Foods. 2023 Mar 24;12(7):1391. doi: 10.3390/foods12071391 (PMC10093910; doi:10.3390/foods12071391)
Supplement: Supplementary file 1 [file foods-12-01391-s001.zip › Supplementary File S1. (a,b).The Principal Scheme of the Experiment .pdf]

Supplementary File S1. (a,b). The principal scheme of the experiment. M – marinades, LM – lamb meat; AW – acid whey; Lc - *Lacticaseibacillus casei* LUHS210; Lu – *Liquorilactobacillus uvarum* LHUS 245; AP – freeze-dried apple pomace; BP – freeze-dried blackcurrant pomace.

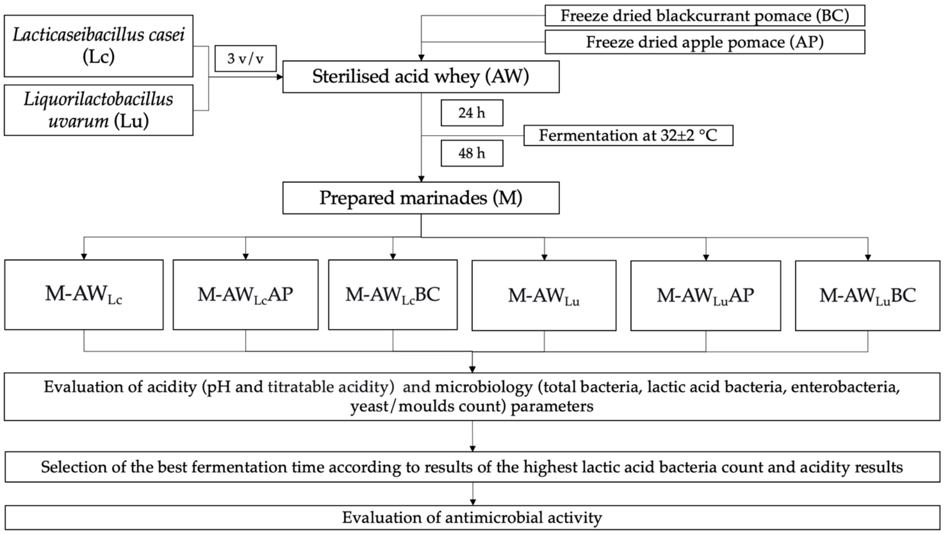

a

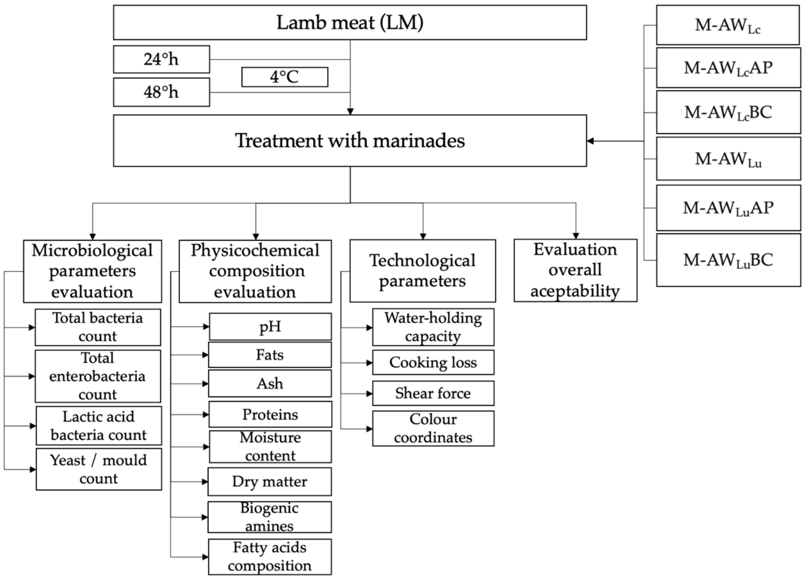

b
