# Supplementary material for: Effects of Marinades Prepared from Food Industry By-Products on Quality and Biosafety Parameters of Lamb Meat
Source: Foods. 2023 Mar 24;12(7):1391. doi: 10.3390/foods12071391 (PMC10093910; doi:10.3390/foods12071391)
Supplement: Supplementary file 1 [file foods-12-01391-s001.zip › Supplementary File S2. Analysis of the Biogenic Amine Content. docx.pdf]

Supplementary File S2.

The chromatographic analyses were carried out using a Varian ProStar HPLC system (Varian Corp.) with two ProStar 210 pumps, a ProStar 410 autosampler, a ProStar 325 UV/VIS Detector, and Galaxy software (Agilent) for data processing. For the separation of amines, a Discovery ® HS C18 column (150 × 4.6 mm, 5 μm; SupelcoTM Analytical) was used. The eluents were ammonium acetate (A) and acetonitrile (B) and the elution program consisted of a gradient system with a 0.8 ml/ min flow rate. The detection wavelength was set to 254 nm, the oven temperature was 40°C, and samples were injected in 20 μl aliquots. The target compounds were identified based on their retention times in comparison with their corresponding standards.
