# Supplementary material for: Effects of Marinades Prepared from Food Industry By-Products on Quality and Biosafety Parameters of Lamb Meat
Source: Foods. 2023 Mar 24;12(7):1391. doi: 10.3390/foods12071391 (PMC10093910; doi:10.3390/foods12071391)
Supplement: Supplementary file 1 [file foods-12-01391-s001.zip › Supplementary File S3. Analysis of the fatty acid profile.pdf]

Supplementary File S3.

The extraction of lamb meat lipids for FA analysis was performed with chloroform/methanol (2:1 v/v), as described by Pérez Palacios et al [58]. Fatty acid methyl esters (FAME) were prepared by esterification with 2 mol/L of KOH in methanol and shaking using a laboratory shaker for 1 hr, upper layer was filtered using a 0.22 µm membrane syringe filter and used for the analysis. The fatty acid composition was determined using a gas chromatograph GC-2010 Plus (Shimadzu Corp.) equipped with Mass Spectrometer GCMS-QP2010 (Shimadzu Corp.). Separation was carried out on a Rxi-5ms column (30 m length, 0.25 mmID, and 0.25µm *df* (Restek)). The mass spectrometer was operated in full scan mode. Analyte was injected in split mode at a 1:60 split ratio. Oven temperature programming started at 40°C, was raised 8°C/min to 220°C, held for 1 min at 220°C, increased again at 20°C/min to 270°C, and held for the last 10 min. Injector temperature was 240°C, interface −270°C, and ion source 220°C. The carrier gas was helium at a flow rate of 0.91 ml/min. The fatty acid methyl esters (FAME) concentration was determined using a calibration curve, and the results were expressed as a percentage of the total FAME concentration in the sample. The calibration curve was prepared using standard Supelco 37 Component FAME Mix (Merck & Co., Inc.). The extraction of lipids was performed once, and all analytical determinations were performed at least in triplicate.
