# Supplementary material for: Effects of Marinades Prepared from Food Industry By-Products on Quality and Biosafety Parameters of Lamb Meat
Source: Foods. 2023 Mar 24;12(7):1391. doi: 10.3390/foods12071391 (PMC10093910; doi:10.3390/foods12071391)
Supplement: Supplementary file 1 [file foods-12-01391-s001.zip › Supplementary Table S1. Fatty acids composition.pdf]

Supplementary Table S1. Fatty acids composition

	C-LM		LM-AW$_{Lc}$		LM- AW$_{Lc}$AP		LM-AW$_{Lc}$BP		LM-AW$_{Lu}$		LM-AW$_{Lu}$AP		LM-AW$_{Lu}$BP	
	**Saturated fatty acids SFAs**													
	**Duration of the treatment, h**													
	24	48	24	48	24	48	24	48	24	48	24	48	24	48
Capric C10:0	0.47± 0.002e	0.45± 0.011f	0.27± 0.010d	0.17± 0.011c	0.08± 0.001a	0.08± 0.002a	0.11± 0.001b	0.12± 0.003b	0.14± 0.002c	0.17± 0.004c	0.28± 0.040d	0.22± 0.008d	0.28± 0.051d	0.30± 0.060e
Lauric C12:0	0.80± 0.110d	0.76± 0.015d	0.55± 0.090c	0.46± 0.040c	0.36± 0.080b	0.14± 0.010a	0.20± 0.040a	0.23± 0.090b	0.17± 0.003a	0.16± 0.010a	0.19± 0.010a	0.29± 0.050b	0.18± 0.009a	0.15± 0.040a
Myristic C14:0	4.14± 0.14a	4.12± 0.18a	5.05± 0.18c	5.06± 0.04b	4.64± 0.13b	4.15± 0.12a	4.45± 0.16b	3.89± 0.09a	3.96± 0.19a	3.83± 0.17a	3.93± 0.12a	4.00± 0.11a	3.91± 0.07a	3.84± 0.04a
Penta-decane C15:0	1.22± 0.09a	1.09± 0.13a	1.21± 0.07a	1.33± 0.100a	1.17± 0.14a	1.25± 0.20a	1.46± 0.15a	1.11± 0.08a	1.31± 0.13a	1.66± 0.03b	1.33± 0.09a	1.21± 0.08a	1.88± 0.10b	1.90± 0.18b
Palmitic C16:0	22.59± 0.75a	22.00± 0.66a	24.54± 0.81a	24.83± 0.53b	24.52± 0.85a	24.43 ±0.45b	24.81± 0.77a	24.97± 0.64b	24.67± 0.54a	23.94± 0.41b	23.28± 0.57a	24.45± 0.69b	23.38± 0.64a	23.32± 0.41b
Stearic C18:0	24.10± 0.43a	23.80± 0.32a	24.07± 0.20a	24.40± 0.25a	24.41± 0.64ab	25.75± 0.31c	24.73± 0.19a	24.83± 0.24b	24.67± 0.29a	24.29± 0.18a	24.64± 0.24a	24.71± 0.37b	24.04± 0.22a	24.18± 0.19a
Margaric C17:0	2.06± 0.12a	2.05± 0.09a	1.91± 0.09a	2.33± 0.16b	1.99± 0.14a	2.13± 0.10ab	2.87± 0.10c	2.80± 0.10c	2.18± 0.17ab	2.30± 0.17b	2.49± 0.17a	2.25± 0.15b	2.37± 0.21b	2.23± 0.14b
Arachidic C20:0	0.33± 0.009b	0.34± 0.070c	0.27± 0.060b	0.20± 0.060bc	0.16± 0.020a	0.06± 0.002a	0.37± 0.081b	0.24± 0.060c	0.22± 0.009b	0.13± 0.013b	0.57± 0.010c	0.24± 0.060c	0.37± 0.015b	0.54± 0.10d
	**Monounsaturated fatty acids MUFAs**													
Palmitoleic C16:1 cis-9	2.82± 0.17b	2.81± 0.11b	1.86± 0.16a	2.13± 0.09a	2.62± 0.23b	2.19± 0.13a	1.97± 0.24a	2.09± 0.10a	2.24± 0.17ab	1.86± 0.14a	2.33± 0.18b	2.17± 0.20a	2.33± 0.11b	2.03± 0.09a
Myristoleic C14:1 n5	0.14± 0.005f	0.11± 0.009b	0.11± 0.003d	0.19± 0.005c	0.13± 0.006e	0.12± 0.004b	0.07± 0.010a	0.07± 0.003b	0.09± 0.003b	0.05± 0.010a	0.15± 0.004f	0.09± 0.020b	0.10± 0.001e	0.12± 0.020b
Oleic C18:1 cis-9	32.45± 0.84a	31.98± 0.72a	32.87± 1.25a	32.82± 0.94a	33.16± 1.02a	33.43± 0.36a	33.78± 0.54a	33.29± 0.47a	32.45± 0.69a	32.45± 0.99a	32.77± 0.88a	33.19± 1.12a	32.74± 0.82a	32.59± 0.93a
Nervonic C24:1	1.67± 0.13c	1.66± 0.21bc	0.83± 0.119a	0.79± 0.080a	0.91± 0.160a	0.94± 0.118a	1.01± 0.09a	0.87± 0.120a	1.37± 0.22b	1.93± 0.24c	1.38± 0.12b	1.35± 0.34b	1.50± 0.15bc	1.36± 0.23b
Elaidic C18:1 n9 trans	0.55± 0.080c	0.59± 0.11b	0.55± 0.072c	0.43± 0.131b	0.27± 0.011b	0.23± 0.090a	0.17± 0.03a	0.29± 0.044a	0.35± 0.069c	0.37± 0.060b	0.45± 0.120c	0.23± 0.044a	0.46± 0.072c	0.63± 0.150b
	**Polyunsaturated fatty acids (PUFAs)**													
Linoleic C18:2 *cis-9,12*	3.70± 0.23b	3.21± 0.11a	3.34± 0.19a	2.84± 0.14a	3.24± 0.16a	2.88± 0.13a	2.83± 0.18a	2.96± 0.22a	3.14± 0.23a	3.45± 0.36b	3.20± 0.30a	2.99± 0.18a	3.64± 0.15b	3.87± 0.18b
α-Linolenic C18:3 *cis-9,12,15*	0.13± 0.030a	0.12± 0.009b	0.12± 0.011a	0.12± 0.003b	0.20± 0.020b	0.29± 0.081c	0.15± 0.003a	0.14± 0.031b	0.23± 0.040b	0.12± 0.004b	0.22± 0.011b	0.05± 0.010a	0.10± 0.030a	0.08± 0.022a
γ-Linolenic C18:3 *cis-6,9,12*	2.49± 0.13c	1.99± 0.12b	2.15± 0.150b	1.44± 0.16a	2.12± 0.144b	1.20± 0.20a	1.14± 0.23a	1.43± 0.13a	2.37± 0.12bc	2.22± 0.23b	2.46± 0.17c	1.85± 0.15b	2.29± 0.19b	2.26± 0.17b
Eicosadienoic C20:2 cis-11,14	0.06± 0.002a	0.03± 0.002a	0.07± 0.022a	0.08± 0.002c	0.13± 0.004c	0.09± 0.020c	0.12± 0.003b	0.05± 0.001b	0.08± 0.011a	0.07± 0.015c	0.10± 0.002a	0.08± 0.020c	0.09± 0.022a	0.05± 0.002b
Arachidonic C20:4 cis-5,8,11,14	0.06± 0.002a	0.05± 0.001a	0.07± 0.021b	0.10± 0.001b	0.19± 0.041c	0.26± 0.041d	0.06± 0.007a	0.12± 0.004c	0.10± 0.010b	0.08± 0.022b	0.06± 0.022ab	0.09± 0.010b	0.07± 0.025b	0.07± 0.01b
Docosahexaenoic C22:6 n3 (DHA)	0.04± 0.002c	0.04± 0.001d	0.03± 0.003b	0.01± 0.002a	0.01± 0.001a	0.02± 0.001b	0.04± 0.002c	0.02± 0.002b	0.03± 0.001b	0.02± 0.002b	0.03± 0.001b	0.03± 0.001c	0.04± 0.011bc	0.07± 0.001e
Eicosapentaenoic C20:5 n3 (EPA)	0.08± 0.002b	0.07± 0.003c	0.05± 0.005a	0.04± 0.010a	0.09± 0.030b	0.08± 0.004d	0.08± 0.010b	0.06± 0.001b	0.08± 0.001b	0.06± 0.002b	0.06± 0.010ab	0.06± 0.009b	0.07± 0.010b	0.05± 0.005a

$^{a-e}$ Mean values with different superscript letters between rows of the same treatment time are significantly different ($p \leq 0.05$); data expressed as the mean value ($n = 3$) ± standard deviation (SD). C – control; LM – lamb meat; AW – acid whey; Lc - *Lacticaseibacillus casei; Lu – Liquorilactobacillus uvarum*; AP - freeze dried apple pomace; BP – freeze dried blackcurrant pomace.
